# Community and healthcare providers’ perceptions of quality of private sector outpatient malaria care in North-western Ethiopia: a qualitative study

**DOI:** 10.1186/s12936-021-03694-2

**Published:** 2021-03-17

**Authors:** Mesele Damte Argaw, Thandisizwe Redford Mavundla, Kassa Daka Gidebo

**Affiliations:** 1 USAID Transform: Primary Health Care Activity, JSI Research & Training Institute, Inc. in Ethiopia, P.O. Box 1392, 1110 Addis Ababa, Ethiopia; 2 Department of Health Studies, University of South Africa (UNISA), Pretoria, South Africa; 3 School of Public Health, College of Health Sciences and Medicine, Welaita Sodo University, Wolaita Sodo, Ethiopia

**Keywords:** Perception, Community, Malaria, Outpatient malaria services, Quality, Public private partnership, Formal private sector

## Abstract

**Background:**

Malaria is one of the most important public health problems in Ethiopia contributing to significant patient morbidity and mortality. Prompt diagnosis and effective malaria case management through public, private and community health facilities has been one of the key malaria prevention, control and elimination strategies. The objective of this study was to evaluate adult malaria patients and healthcare providers’ perception of the quality of malaria management at private sector outpatient facilities.

**Methods:**

An exploratory, descriptive, contextual and qualitative research methodology was conducted with 101 participants (33 in-depth interviews (INIs) and ten focus group discussions (FGDs) with 68 participants). All interview and focus group discussions were audio recorded, transcribed verbatim and analysed, using eight steps of Tesch.

**Results:**

During data analysis a single theme, two categories and six sub-categories emerged, namely (1) perceived quality of malaria management at outpatient facilities; (a) essential resources; (a1) safe outpatient services; (a2) anti-malarial drugs and supplies; (a3) health workers; (b) factors influencing service utilization; (b1) physical accessibility; (b2) “art of care’’; and (b3) efficient malaria diagnosis and treatment services. Both FGDs and INIs participants had a positive perception of the quality of malaria outpatient services at private health facilities. The positive perceptions include safe and clean facility; availability of supplies and comprehensive services; convenient working hours; short waiting hours and motivated, competent and compassionate health workers. However, some participants raised their safety concerns due to perceived poor infection control practices, small working areas, interruption of anti-malarial supplies and inefficient malaria diagnosis and treatment services.

**Conclusion:**

Both community members and healthcare providers had more positive perceptions towards outpatient malaria services offered at private health facilities. However, positive behaviour must be maintained and concerns must be dealt with by enhancing functional public private partnership for malaria care services to improve private sector malaria case management; build the service providers’ capacity; ensure uninterrupted anti-malarial supplies and empower the community with early health-seeking behaviour.

## Background

Malaria is an entirely preventable and treatable parasitic disease [1]. In the last two decades, substantial progress has been made with the fight against malaria [2]. According to the latest estimates of the World Health Organization (WHO) between the year 2000 and 2015, the global malaria case incidence was reduced by 41% and the malaria-related death rate by 62% [3]. However, malaria continued to be a major global public health concern. In 2018, malaria accounted for 228 million cases and 405,000 deaths [4].

In Ethiopia, just like in other parts of sub-Saharan Africa (SSA), malaria is the major public health problem affecting 75% of the 1.1 million square kilometre land mass, where over 60% of 99 million people lived at risk of acquiring the disease in 2015 [5, 6]. More specifically 14.0% (104, 202/743,851) of reported outpatients were malaria cases in the West Gojjam Zone in 2015. This figure contributed to the second largest number of malaria cases among the ten zones of Amhara region, Ethiopia [7].

It is recommended that access to universal parasitological diagnosis and prompt treatment with effective anti-malarial drugs be enhanced for confirmed cases through public health facilities, private health facilities and community-level services [5, 8]. Although the public health sector is a major health services provider in many malaria endemic countries, the private health sector provides services for a significant number of malaria and non-malaria fever management [8–11].

Currently, the private health sector in Ethiopia is regarded as an untapped potential to improve the quality of healthcare services. The country’s health policy over the last 24 years demonstrates the government’s commitment to the sector as evidenced by the increase in the number of private health facilities to about 7304 (75 hospitals 19 health centres and 7210 clinics) in the year 2015 [12–14]. But an increased number of health facilities might not be enough to ensure provision for quality malaria diagnosis and treatment services. In addition, the perception of community and healthcare providers of the quality of services have a direct impact on clinical decision, utilization of quality malaria diagnosis and treatment services [15]. Therefore, engagement of healthcare providers, patients or caretakers improves access to affordable standardized public health services and the demand for it. However, the perceptions of both healthcare providers and adult outpatient service beneficiaries of the quality of malaria care had never been explored and described in Ethiopia [16]. Hence, the aim of this study was to explore and describe the perceptions of adult malaria outpatients and healthcare providers of the quality of malaria management at private health facilities in four districts, namely: Finote Selam, Jabih Tehina, Bure and Womberma of West Gojjam Zone, North-western Ethiopia.

### Definition of key concepts

#### Medium private clinic

According to the Ethiopian standards [17], a medium private clinic should be led by personnel who achieved educational level either of Medical Doctor or Public Health Officer or Bachelor of Science in Nursing; and a medium clinic should have a minimum of four additional health personnel—two diploma nurses and two laboratory technicians to run functional preventive, curative and rehabilitative services.

#### Private

The word denotes two sets of structures; the private -for-profit encompassing commercial enterprises of any size and the private for-non-profit referring to the non-governmental organizations (NGOs), philanthropic entities and others not for profit [18]. In addition, private providers are those that fall outside the direct control of government [19].

#### Private health sector

Comprises all providers who exist outside the public sector, whether their aim is philanthropic (not-for-profit) or commercial (for profit) to treat illness or prevent disease [18].

#### Private health facilities

Are health facilities owned by individuals who seek to earn profit; clinics and hospitals owned by private employers; those operated by religious missions and other non-governmental organizations (NGOs) [19]. In this research private means health facilities established for profit, which provide healthcare services based on a user fee and include medium clinics.

#### Outpatient facility (ambulatory setting)

A type of institutional organized health setting in which health services are provided on an outpatient basis [20]. An outpatient is a patient who is not hospitalized for 24 h or more but who visits a hospital, clinic or associated facility for diagnosis or treatment [21]. Treatment provided in this fashion is called ambulatory care. In this research outpatient facility means that all malaria diagnosis and treatment services are available at selected private facilities for ambulatory patient management.

#### Uncomplicated malaria

“Symptomatic malaria without signs of severity or evidence vital organ dysfunction. The signs and symptoms of uncomplicated malaria are non-specific. Malaria is, therefore, suspected clinically mostly on the basis of fever or a history of fever” [22].

#### Focus group discussion (FGD)

Is four to ten respondents brought together to discuss the research topic as a group [23]. The purpose and design of FGDs include obtaining the participants’ perceptions in a focused area in a setting that is permissive and non-threatening [24]. For this research, the researcher facilitated ten FGDs. Moreover, the group dynamics and respondent behaviours were documented with hand-written notes.

#### Interview

Involves verbal communication during which the study participants provide information to the researchers [25]. A qualitative researcher would get the opportunity for a detailed understanding of deeply rooted personal contexts in which the phenomenon is located [23].

## Methods

### Study design

An exploratory, descriptive, contextual, qualitative study was conducted between October 2016 and January 2017[16, 25].

### Study area

The Amhara region where this study was conducted is one of the nine administrative regional states and two city administrations of the Federal Democratic Republic of Ethiopia. West Gojjam Zone is one of the ten administrative zones of the Amhara region. It covers an area of 13,669 square kilometres; and the zone is further divided into 18 *woredas* (districts). *Woreda*, or district is an area delineated as the basic unit of planning and political administration at a lower level with a population of between 60,000 and 100,000; and further subdivided into the lowest government administrative units known as *kebeles* (villages) [5]. Based on the 2007 National Population Census, the West Gojjam Zone had a projected population of 2,517,825 million people in the year 2015 and 87% of them were rural residents [26]. Eleven medium clinics are providing malaria diagnosis and treatment service in the study areas. All medium clinics are established as private for profit organizations, but six out of 11 facilities are serving the community through public private partnership (PPP) for malaria care services, where partner facilities had access for anti-malarial supplies and received technical support from public health sector.

### Population and sampling strategy

Adults older than 18 years who were outpatient malaria service beneficiaries and healthcare providers working at the 11 targeted medium clinics in the Finote Selam, Jabih Tehina, Bure and Womberma districs, West Gojjam Zone, North West Ethiopia.

The West Gojjam zone was selected with purposive sampling for its accessibility to the main road and convenience to researchers. However, districts were selected based on the high burden of malaria. In the year 2012, the incidence of malaria in the selected four districts ranged from 40 to over 100 per 1000 population/November 2012 [27]. All 11 medium clinics were enrolled in this study.

### Data collection

Data were collected through in-depth individual interviews (INIs), FGDs and field notes. In-depth interviews were conducted at their workplace with 33 healthcare providers, using an iterative process. In average, each in-depth interview lasted 45–60 min. In addition, data were collected from ten focus groups (five men and five women) by facilitating discussions in the hall of farmer training centre with 68 adult outpatient services beneficiaries who were diagnosed and treated for uncomplicated malaria infections. On average, each FGD lasted one and a halve hour. Sixty-eight patients who voluntary consented to participate in this study were identified with their permanent address and reminded twice to participate. Each FGD consisted of 6–8 participants. Furthermore, handwritten notes were taken by observing patient and health workers’ interaction; and assessing the outpatient facilities. Those patients who had completed their anti-malarial treatment prior to the data collection were used for the FGDs. The data collection was ceased based on saturation or redundancy of data [28].

### Data analysis

With the qualitative approach, the researcher analysed the data inductively to build from particular to general theme and interpret the meaning of the data [29]. The researcher followed the data analysis process by performing the eight steps: (1) to get a sense of the whole by reading all transcripts (2) picking one on the top of the pile, reading the transcript and re-reading it; jotting down ideas in the margin on each page; (3) after completing the second step for several files, the researcher listed topics into columns; and the topics were abbreviated as codes; (4) the researcher took the list, reviewed the data and read the abbreviated codes over against the text; then performed preliminary analysis; organized the arrangement to see if new categories and codes emerged; (5) selected most describing words or categories; studied the data for internal convergence and external divergence; (6) made a final decision on each category and alphabetized the codes; (7) assembled the data material belonging to each category and performed preliminary analysis; and (8) Furthermore, the raw data were re-coded by experienced qualitative researcher and consensus discussions were arranged on the theme. During data analysis, a single theme, two categories and six sub-categories were identified [29].

### Measures for ensuring trustworthiness

The four criteria for trustworthiness: truth value, applicability, consistency and neutrality were ensured throughout the process [30, 31]. Rigors were ascertained through the concepts of qualitative paradigm and translated through prolonged engagement, triangulation, peer debriefing, members check and dense description. In addition, credibility, transferability, dependability and conformability have been used to describe various aspects of trustworthiness [31, 32]. See Table 1 below for applicability of these criteria in this study.

**Table 1:** Measures for ensuring trustworthiness

Strategy	Criteria	Applicability
Credibility	Prolonged engagement	The researcher served as malaria programme manager for over six years; in addition, in this study during data collection, the researcher spent more than three months studying in this area
	Triangulation	The data were collected, using semi structured INIs, FGDs and observations
	Peer debriefing	A sociologist with qualitative research expertise recoded the data and consensuses were reached on the theme, categories and subcategories
	Member checking	During data collection, the researcher verified the information of interviewees through paraphrasing. During analysis emerging themes and categories were shared with research participants; and finally, the result was shared with research participants in the study area
Transferability	Dense Description	The research presented the context with dense description; and detailed presentation of the result with verbatim transcripts
Dependability	Dependable audit	A sociologist recoded the data
	Triangulation of data	Data were collected, using an audio tape recorder, typed verbatim transcripts, field handwritten notes and observation
	Dense description	Detailed description of methodology was presented
Conformability	Triangulation	Triangulation of the findings of the researcher and independent research expert was considered

The table presents summary of measures taken to ensure trustworthiness

### Ethical considerations

The study was approved by the Institution Review Board (IRB) of the University of South Africa (UNISA) and Amhara Regional State Health Bureau, Research and Technology Transfer Core Process. The supporting letter was obtained from the West Gojjam zone Heath Department and the Finote Selam, Jabih Tehina, Bure and Wonberma district health offices. Additionally, all participants were informed of the overall purpose of the study, methods of data collection, benefits and risk of participation, confidentiality and their right to withdraw anytime during the INIs and FGDs and estimated time to complete the task. After receiving information all study, participants voluntarily signed the consent form. To maintain the confidentiality of collected data, anonymity was maintained throughout the research process [33,34].

## Results

In total, 10 FGDs were facilitated among 68 adult male and female outpatient malaria service beneficiaries at the private health facilities in the West Gojjam Zone, Ethiopia. In addition, 33 INIs were conducted with private healthcare providers.

### Demographic characteristics

Table 2 presents, the demographics of the participants. Both male and female community members participated in the FGDs. Almost half 33(48.5%) of the participants were female. The age of the FGD participants ranged between 20 and 59 years (median 26). Most participants were married. Two thirds of the INIs participants were male, and their age ranged between 23 and 58 years (median 32). One third of INIs participants were nurses by profession.

**Table 2:** Demographics of community members and healthcare providers

Variables	Community members (n = 68)	Health care providers (n = 33)
Gender		
Male	48.5% (33/68)	69.69% (23/33)
Female	51.5% (35/68)	30.03% (10/33)
Age (in years)		
Range	39	35
Median	26	32
Mean	27	33
Marital status		
Single	25.0% (17/68)	30.3% (10/33)
Married	75.0% (51/68)	69.7% (23/33)
Education		
Read and write	20.6% (14/68)	NA
Primary	17.7% (12/68)	NA
Secondary	33.8% (23/68)	NA
College (Diploma /10 + 3)	27.9(19/68)	60.7% (20/33)
Bachelor’s degree (12 + 4)	NA	33.3(11/33)
Master’s degree (12 + 6)	NA	6.0% (2/33)
Employment		
Unemployed	1.5% (1/68)	NA
NGO	4.4% (3/68)	NA
Student	7.4% (5/68)	NA
Farmer	11.8% (8/68)	NA
Private business	16.2% (11/68)	NA
Housewife	20.6% (14/68)	NA
Public servant	38.2% (26/68)	NA
Profession		
Nurse	NA	33.3% (11/33)
Laboratory Technician	NA	30.3% (10/33)
Public Health Officer	NA	30.3% (10/33)
Medical Doctor	NA	6.1% (2/33)

The table presents the socio-demographic characteristics of the study participants healthcare providers

### Perceived quality of outpatient malaria services

This theme featured the perception of patients and health workers of the quality of private health facilities and outpatient malaria care services. Two categories were revealed in this theme, namely: (1) essential resources and (2) factors influencing utilization of outpatient services (Table 3).

**Table 3:** Theme, categories and sub-categories emerged from qualitative data analysis

Perceived quality of outpatient malaria services	Categories	Sub-categories
	Essential resources	Safe outpatient facilities
		Anti-malarial drugs and supplies
		Health workers
	Factors influencing the utilisation of outpatient services	Physical accessibility
		“Art of care’’
		Efficient malaria diagnosis and treatment services

The table presents a single theme, two categories and six sub-categories of the qualitative data

### Essential resources

In this category, the researcher identified three sub-categories: (1) safe outpatient services; (2) anti-malaria drugs and supplies; and (3) health workers.

### Safe outpatient facilities

The majority of healthcare providers perceived that the private sector’s outpatient malaria care service facilities were safe for patients /and caretakers. In addition, they perceived the quality of outpatient care for malaria patients in the private medium clinics in terms of better facilities and water supply and electric power supply and experiences of healthcare providers. The following verbatim quote clearly explains the health workers’ perceptions of safe and comfortable waiting areas:

“The waiting area in my clinic is adequate and safe, equipped with comfortable seats, and video shows.” [In-depth Interview: HF2, HW1].

Another two health workers made the following statements about safe outpatient facilities with their good biohazard management:

“Private clinics have piped water supplies, and uninterrupted electric power sources which are essential for cleaning and sterilizing medical equipment.” [In-depth Interview: HF2, HW1]

“My clinic has a fence to protect access to bio-medical hazard by humans and animals, with a clean compound and it is safe for patients or to their attendants and community at large.” [In-depth Interview: HF11, HW1]

A participant in the in-depth individual interviews explained the perception of the quality of outpatient services regarding the availability of competent and experienced health workers in the following manner:

“In our health facilities, all of us [health workers] have a valid professional licensure from Ethiopian Food, Medicine and Health Care Administration Authority; we can provide safe services in the national health system.” [In-depth Interview: HF1, HW1]

“[name] private clinic hires experienced health workers.” [In-depth Interview: HF10, HW2]

In addition, the focus group participants perceived private outpatient malaria facilities to be well-kept, small health facilities that have a few well-labelled rooms that enable patients to easily walk to obtain safe outpatient malaria services. The following verbatim statement was articulated by one of treated adult malaria patients who used private sector outpatient services:

“Unlike hospitals or health centres, private clinics have few rooms with labelled signs; I can easily reach where I want to go whether it is laboratory or bath or injection rooms.” [FGD2: Participant M (2)]

On the other hand, a few healthcare providers and some malaria outpatient service beneficiaries stated their safety concerns and a higher risk of acquiring diseases through the poor quality of outpatient malaria services at private facilities. One female focus group participant raised the following safety concern:

“The majorities of the auxiliary staff, who work in the private clinic, are not able to be employed within the public health sector, and they may not pass the national exam prepared by the centre of excellence.” [FGD5: Participant F (1)]

In-depth interview participants elaborated on their safety concerns in relation to facilities for service provision by stating the following:

“My laboratory room size is too small compared to the nationally recommended standard of 20 square metres; it is difficult to provide safe services.” [In-depth Interview: HF4, HW3]

“Almost all private clinics are constructed for individual housing; it is difficult to make it a standard health facility.” [In-depth Interview: HF5, HW1]

#### Anti-malarial drugs and supplies

In this study, almost half of the targeted facilities had a signed memorandum of understanding (MoU) with/between Town Health Office, Zone Health Department and the Regional Health Bureau to work with Public Private Partnership for malaria case management. This legal relationship enables partner private health facilities to obtain anti-malarial drugs and supplies and receive technical support. Most healthcare providers perceived frequent stock-out, interruption of supplies; a lack of reliable laboratory supplies to seriously affect the quality of their malaria outpatient services. The following verbatim statements were made on the stock out and interruption of anti-malarial drugs as a perceived challenge to provide high-quality outpatient malaria services by a healthcare provider:

“….. health facility engaged in Public Private Mix Partnership for malaria care services, though we have a valid Memorandum of Understanding with the District Health Office, Zone Health Department and Regional Health Bureau, our service quality was seriously affected by frequent stock out and interruption of antimalarial drug supplies. This unreliable access to supplies has some harm on our reputation. Our clients feel as if we do not want to give them the drug while it is their right to get antimalarial drugs free of charge within our facility.” [In-depth Interview: HF2, HW3].

Healthcare providers from HF8 and HF10 perceived that a shortage and lack of quality-assured laboratory supplies impacts on the quality of outpatient malaria services:

“There is a shortage of laboratory supplies; I couldn’t get absolute methanol which is useful to fix thin blood films. Therefore, I use to work with less reliable methods to report accurate malaria parasite species and quantify using thick blood film. This is not in agreement with the recommendations of the National External Quality Assurance (EQA) guidelines, which states thick film for screening for the presence of the parasite in blood and the thin film to identify species and quantify the parasite load.” [In-depth Interview: HF8, HW2]

On the other hand, treated malaria patients clearly depicted that the availability of various anti-malaria drugs and laboratory services attract them to regularly visit private clinics:

“If you visit public health facilities, you do not get either the laboratory service or the drugs, while in private health facilities there are a number of antimalarial drugs…” [FGD1, Participant F (3)]

“I went to the public facilities, there were no drugs, and I got the necessary drugs from private facilities.” [FGD5, Participant F (5)]

#### Health workers

The majority of healthcare providers and patients perceived the high-quality outpatient malaria services in line with the availability of experienced and competent healthcare providers at the targeted health facilities. Some healthcare providers reported that they are committed to quality services by hiring experienced healthcare providers to exceed beyond the expectations of their customers. One healthcare provider from HF2 made the following statement:

“Our patients expect high-quality services from us; we always prepared ourselves to exceed their expectations… we used to hire experienced health workers.” [In-depth Interview: HF2, HW1]

In addition, treated malaria patients explained their perceptions based on their most recent visits to private health facilities as healthcare providers were working hard to meet their expectations. The following statement illustrates the experience of one of the adult malaria patients:

“I am one of the regular customers of this [name] clinic for over 12 years; the facility has experienced specialist doctors, nurses and laboratory professionals… all were working hard to identify my health problem and treats me in a good manner.” [FGD2, Participant M (5)]

However, some FGD participants reported that they received poor quality healthcare services by junior and inexperienced health workers:

“…in private health facilities, we used to visit to be examined by highly experienced health workers, mostly owners or managers of the health facility. However, there are junior and non-experienced health workers within the team.”

“…the health worker took blood sample from my fingertips but she didn’t give me at least clean cotton to hold on the bleeding site…” [FGD1, Participant F(1)]

### Factors influencing the utilization of outpatient services

Most patients elicited their positive perceptions of the outpatient malaria care services they received from private health facilities. There were, however, some patients who reported their concerns about the availability of effective drugs and competent health providers. The following three sub-categories emerged: (1) physical accessibility; (2) “art of care’’; and (3) efficient malaria diagnosis and treatment services.

### Physical accessibility

The majority of healthcare providers reported their service quality in terms of convenience in opening hours, located within a short distance for the community and offered with affordable cost for the general public. The following statements were made by the health workers:

“The service hour is convenient for all our clients. We are open for about 16 to 18 h per day seven days a week.” [In-depth Interview: HF8, HW 1]

“My customers come from both the rural and urban area of West Gojjam zone and our service cost is affordable for our customers, otherwise we couldn’t stay in business.” [In-depth Interview: HF2, HW1]

In addition, malaria patients elaborated as they received accessible malaria services without any discrimination. The following transcripts from FGD10 clearly show the existence of equal treatment among service beneficiaries at targeted health facilities:

“In private health facilities they equally served their customers; whether you are poor or rich; urban or rural dwellers; wear clean or dirty clothes; literate or illiterate.” [FGD10, Participant M (4)]

“In my district, access to malaria treatment is high, and can get service within one hour; the private health facilities do not give priority to urban dwellers, and better off. Since we all get the service with service fee out of our pocket, everybody equality treated by health providers.” [FGD10, Participant M (1)]

In addition, two FGD participants elaborated that accessibility of services was not deterred by the distance of health facility from their home:

“I come here within 10 min of walk on foot; I am very close to the [name] clinic” [FGD9, Participant F (5)]

“My home is very far; it is about 50 kms away from here [name] but my children bring me here for better quality of services.” [FGD10, Participant M (7)]

### “Art of care’’

Most private healthcare providers had the art of care, which is compassionate, respectful and caring by nature. The healthcare providers believe that the private health sector would not survive in business if they were not responsive to patients’ needs and demands in many ways. The following statement echoes the sentiments of the majority of in-depth interview respondents:

“I always greet my patients and ask about their families and community, and then I respectfully listened to their health problems. I also try to express my empathy and feeling properly. Finally, I encourage them to participate in selecting their treatment.” [In-depth Interview: HF1, HW3]

“Every time, when patients get into my clinic, I used to screen for life threatening situation and the second thing I do is alleviating severe pains. Then, I will take detail medical history, perform a physical examination and order selective laboratory investigations. Finally, I try to involve the patient in the management plan for their identified health problems.” [In-depth Interview: HF10, HW1]

The adult malaria patients from FGD had the following to say about fast, respectful, caring service:

“The service I received in the [name] clinic was fast, the health workers were showing me their respect and take care of me throughout the process.” [FGD8, Participant M (8)]

### Efficient malaria diagnosis and treatment services

Two individual in-depth interview respondents from HF3 and HF7 made the following statements:

“Our patient wants over treatment; we are not efficient in using limited resources. We used to prescribe antimalarial drugs, with vitamins, dextrose, antibiotics etc.…” [In-depth Interview: HF3, HW3]

“If my patient is negative for malaria, I will repeatedly check before I declared fever with malaria unlikely …” [In-depth Interview: HF7, HW2]

Adult malaria patients stated that the service they received from private health facilities’ outpatient malaria services were not efficient in diagnosis and treatments, as can be seen from the following statement by one of the FGD1 participants:

“I received treatment for malaria, typhoid and typhus fever. I took over four drugs: Coartem, Ciprofloxacin, Doxycycline, and Paracetamol. The prescription I collected from the clinic was full of text from head to toe.” [FGD1, Participant F(3)]

## Discussion

The perceptions of health workers and customers regarding outpatient services have a positive and negative effect on clinical decisions, utilization of services and patients’ compliance with healthcare providers advices [15, 35, 36]. This exploratory and descriptive qualitative study generates information for policymakers, service providers and programme managers to enhance quality malaria outpatient services through evidence-based decision-making on private health sector. Both FGDs and INIs participants had a positive perceived quality of malaria outpatient services in terms of the availability of safe and clean facilities, better supplies and comprehensive services, convenient working hours, motivated, competent and compassionate health workers. However, some participants raised their safety concerns due to their perceived poor infection control practices, small working areas, interruption of anti-malarial supplies and inefficient malaria diagnosis and treatment services.

According to the participants, the structural component of outpatient facilities of the private health sector fulfilled their desires and expectations to receive quality management of malaria. The participants perceived the enrolled private health facilities as clean rooms with labels, comfortable waiting areas and availability of comprehensive services. In addition, most healthcare providers believed that their health facilities were safe for patients, attendants and the community at large. Patient safety, freedom from unnecessary harm or potential harm associated with health care is the minimum standards which need adherence by all actors [8, 22, 37]. A study conducted at 185 private health facilities in Ethiopia, attests that almost all were equipped with basic amenities useful to comfort patients [38]. However, some healthcare providers were not satisfied with the narrow working rooms; and most private health facilities were constructed as a living house which makes it difficult to provide standard management of malaria outpatients. This finding was in line with findings that health laboratory services in SSA are grossly compromised by poor infrastructures [39].

The FGD participants had a better perception of the availability of anti-malaria diagnostics and treatment supplies at private health facilities than public health facilities. This finding was consistent with the result of INIs among healthcare providers where they described their efforts made in exceeding beyond the expectations of private health facility service beneficiaries. Studies conducted in Nigeria and Ethiopia documented that patients used to visit private health facilities for availability of better-quality diagnosis services, essential drugs and other supplies [35, 40].

In any health system, the presence of qualified, motivated and competent human resources is essential for providing quality health services to patients [41–44]. Most FGD participants expressed their opinion about the availability of experienced healthcare providers. Similarly, the INIs participants expressed the availability of licenced and experienced healthcare providers at private health facilities. This could be due to the minimum standards set by the regulatory bodies. In addition, patients would prefer private health service providers for its continuity of care with a single healthcare provider. This finding was in line with the argument that in the competitive world it is crucial for the success or survival of an organization to meet or exceed patient expectations [45].

In this study, both healthcare providers and patients had positive perceptions of better accessibilities to the management of malaria outpatients in the private health sector. This finding was in line with the WHO’s criterion of access to health services, which consists of a healthcare that is timely, geographically reasonable and provided in a setting where skills and resources are appropriate to medical needs [44]. In addition, the majority of uncomplicated malaria patients sought management of malaria at outpatient facilities of the private health sector to avoid delays. This could be owing to some conveniences offered by the private health sector to customers, as stated by the participants and include short lines, reasonable waiting times and convenient service hours. This finding was in line with that of the majority of private sector malaria patients who were satisfied with the short waiting times for diagnosis services [46].

In this study, the patient-provider interaction was found with exemplary levels of the “Art of Care”. The participants perceived the quality of malaria management they received from respectful and caring professionals. The healthcare providers reported that they used to greet patients politely; worked hard to alleviate pains; gave the opportunity to participate in decision-making; asked questions and offered patients a set of health messages. This finding was in line with the recommended practice towards interest to alleviate pains and facilitate proactive communication between healthcare providers and patients [47, 48]. Responsiveness is also one of the key components of person-centred care which includes compassion, dignity and respect [47–49]. Similarly, a study conducted in a referral hospital of Amhara region, confer the welcoming attitude to clients; the offering of seats to patients; effective communication and patience to clients as some of characteristics of quality of healthcare services [50].

The participants revealed that healthcare providers used to diagnose and treat patients through inefficient ways. Unlike the national and international recommendations of investigating patients with think and thick blood film for microscopy, most febrile patients were investigated for malaria using rapid diagnostic tests or only thick blood film. This finding was in line with that the lack of supplies was the main reason for the poor quality of malaria diagnosis services [51]. In contrast, the most efficient and gold standard malaria diagnostic method in a medium clinic is quality-assured malaria microscopy [11]. In addition, uncomplicated malaria cases were treated with three or more drugs. This could be explained by an antibody based on the common diagnosis practice of typhoid fever and typhus fever; and the private health providers may overtreat patients on their demand. This finding was contrary to the WHO’s recommendation on efficient health service which strived to maximize resource use and avoid waste [44]. However, the finding was in line with a retrospective clinical audit confer that only one fifth of malaria patients had received ideal anti-malarial prescriptions in line with the local and international recommendations. In Angola, there was a wide range from one-third up to half of uncomplicated malaria patients who received services from health workers with poor adherence to guidelines [52].

## Strengths and limitations

Ethiopia has launched a malaria elimination strategy since 2017. Prompt diagnosis and treatment is the main strategy for early identification of cases and to cease transmission. This study identifies the perceptions of community members and healthcare providers regarding the quality of malaria outpatient services offered at one of the service delivery points, that is, the private health facilities. The study has some limitations. Since all participants were enrolled at private health facilities, the study lacks the opinion of a similar group of participants from the public health sector. In addition, this study targeted only adult malaria outpatient service beneficiaries in a zone of the Amhara region. Hence, the generalizability and interpretation of results should consider the stated limitations.

## Conclusion

This qualitative exploratory and descriptive study was conducted in the West Gojjam Zone of Amhara Region, North West Ethiopia. The result of the study suggests that both adult malaria outpatient service beneficiaries and health providers had positive perceptions towards quality outpatient malaria services at private health facilities. However, there are areas of improvement in terms of ensuring the safety of patient and healthcare providers, interruption of anti-malarial drugs and supplies, poor quality of laboratory reagents and inefficient management of malaria patients.

Based on the results of this study the following recommendations were made to improve the quality of outpatient malaria service through enhancing functional public private partnership to improve quality of malaria case management: (1) to use the potential and capacity of the private sector through collaboration; (2) providing tailored capacity building to private health sector providers through case management trainings, coaching and mentorship; (3) empowering the community to seek medical care in advance, using targeted Social and Behavioural Change Communication strategies; and (4) future research on effectiveness of Public Private Partnership is recommended.

## Data Availability

The datasets used and/or analysed during the current study are available from the corresponding author on reasonable request.

## Abbreviations

EFMOH: Ethiopian Federal Ministry of Health
FGD: Focus group discussion
NGO: Non-governmental organization
PPPs: Public–private partnerships
SSA: Sub-Saharan Africa
UNISA: University of South Africa
WHO: World Health Organization
